# High-Affinity Dkk1 Receptor Kremen1 Is Internalized by Clathrin-Mediated Endocytosis

**DOI:** 10.1371/journal.pone.0052190

**Published:** 2012-12-14

**Authors:** Sanjay K. Mishra, Lacey Funair, Alex Cressley, George K. Gittes, R. Cartland Burns

**Affiliations:** 1 Department of Pediatric Surgery, Children’s Hospital of Pittsburgh, Pittsburgh, Pennsylvania, United States of America; 2 Division of Pediatric General and Thoracic Surgery, Nemours Children’s Hospital, Orlando, Florida, United States of America; Northwestern University Feinberg School of Medicine, United States of America

## Abstract

Kremens are high-affinity receptors for Dickkopf 1 (Dkk1) and regulate the Wnt/β-catenin signaling pathway by down-regulating the low-density lipoprotein receptor-related protein 6 (LRP6). Dkk1 competes with Wnt for binding to LRP6; binding of Dkk1 inhibits canonical signaling through formation of a ternary complex with Kremen. The majority of down-regulated clathrin-mediated endocytic receptors contain short conserved regions that recognize tyrosine or dileucine sorting motifs. In this study, we found that Kremen1 is internalized from the cell surface in a clathrin-dependent manner. Kremen1 contains an atypical dileucine motif with the sequence DXXXLV. Mutation of LV to AA in this motif blocked Kremen1 internalization; as reported previously for other proteins, the aspartic acid residue in Kremen1 is not critical. Inhibition of expression of the adaptor protein 2 (AP-2) or inhibition of clathrin by pitstop 2 also blocked Kremen1 internalization. The novel amino acid sequence identified in Kremen1 is similar to the motif previously identified in hydra, yeast, and other organisms known to signal from the trans-Golgi network to the endosomal compartment.

## Introduction

Cell signaling networks allow cells to proliferate and differentiate based on environmental conditions. Molecular events occurring at the plasma membrane surface are organized by a number of well-defined transmembrane modular receptor proteins. Internalization of cell surface transmembrane proteins is governed by the presence of sorting motifs in the cytosolic tail of the transmembrane protein [1], [2]. Some of these sorting motifs are recognized by adaptor proteins present on the membrane facing clathrin-coated pits [1], [2].

The Wnt/β-catenin signaling pathway is often required in cellular events such as proliferation, differentiation, migration, and determination of polarity [3]. Wnt induces Frizzled and its co-receptor low density lipoprotein receptor-related protein 6 (LRP6) to heterodimerize, and this activates the canonical multifunctional β-catenin signaling cascade. Kremens are high-efficiency receptors of Dickkopf 1 (Dkk1), a protein that destabilizes Wnt/β-catenin signaling. Like Wnt, Dkk1 binds to LRP6. Dkk1 and LRP6 form a ternary complex with Kremen2 [4]. Kremen is internalized via clathrin-mediated endocytosis in a route similar to that observed for other endocytic receptors solely dependent on clathrin for trafficking [5]. Caveolin is also necessary for Wnt-dependent internalization of LRP6 [5]–[7]. It was previously suggested that formation of a ternary complex by Dkk1, LRP6, and Kremen negatively regulate Wnt/β-catenin signaling [4].

Clathrin-mediated endocytosis (CME) is a hallmark of all mammalian cells; the process occurs constitutively and continuously [8]. CME plays a significant role in developing organs by maintaining or clearing activated signaling receptors [9]. In CME, clathrin is polymerized by endocytic adaptors such as AP-2, epsin, or Dab2 depending on the type of receptors to be internalized [10]–[12]. Five different types of heterotetrameric adaptor proteins (AP-1, 2, 3, and 4) have been identified [13], [14]. Of the five, AP-2 is a key component of clathrin-coated pits. AP-2 is composed of two large, approximately 100 kDa subunits (α and β2), one 45-kDa medium chain µ2-subunit, and one 23 kDa small σ2-subunit. AP-2 forms an α−σ hemicomplex and a β2−µ2 hemicomplex to form a heterotetrameric complex [15]. The AP-2 recognizes the short linear sequences present on the many receptors facing the cytosolic region. The first sorting signal identified was a dileucine-type sorting motif and dileucine-based sorting motifs such as DXXLL or [D/E]XXXL[L/I] are recognized by the membrane bound adaptor protein AP-2 in the clathrin-coated vesicles. Unlike the tyrosine-based sorting signal YXXφ, which binds to the µ2 subunit of AP-2, dileucine-based sorting motifs binds to the α−σ2 hemicomlex for their sorting [2].

It remains to be illustrated whether YXXφ, [D/E]XXXL[L/I] and FXNPXY motifs make up the entire collection of sorting motifs for clathrin-mediated endocytosis, or whether another variant of these signal sequences could also participate in sorting events. Here, in this study, we would like to decipher the required sequence in Kremen1, which helps in its internalization in a clathrin-dependent pathway.

## Materials and Methods

### Plasmids and Constructs

The mouse Kremen1 cytosolic tail (414–473 amino acid) was amplified from E13.5 mouse brain and ligated into Tac-pCDNA (kind gift from Rebecca Hughey, University of Pittsburgh, Pittsburgh PA, USA) between XhoI and NotI sites. Full-length human Kremen1 was obtained from the ATCC and cloned into pCMV-Tag 4.1 or pcDNA3-mRFP was a kind of Dr. Doug Golenbock (Addgene plasmid # 13032). LRP-6-YFP (kind gift from Dr. Christoph Niehrs, German Cancer Research Center, Heidelberg, Germany). Dkk-1-Flag in pCS2 was a gift of Dr. Xi He (Addgene plasmid #16690), clathrin LC-eYFP was a gift of Dr. Xiaowei Zhuang (Addgene plasmid #21741), and caveolin-GFP was a gift of Dr. Ari Helenius (Addgene plasmid #14433). Mutagenesis was performed by using Agilent Quickchange mutagenesis PCR kit. Sequences of all plasmids were confirmed by sequencing.

### Reagents and Antibodies

Monoclonal antibody anti-Tac was obtained from Ancell Laboratories, and mouse anti-Tac was obtained from Novus. Mouse monoclonal anti-Flag was obtained from Sigma-Aldrich. Rabbit anti-CD71 transferrin receptor antibody was obtained from Novus. An anti-α subunit AP-2 antibody was obtained from Santa Cruz Biotechnology. Anti-AP-2 GD/1 and clathrin heavy chain TD.1 (kind gifts from Linton Traub, University of Pittsburgh, Pittsburgh, PA, USA). Transferrin conjugated with Alexa488 was obtained from Invitrogen.

### Cell Culture and Microscopy

Human embryonic kidney 293T **(**Hek 293T) cells were obtained from American Type Culture Collection and HeLa SS6 cells were grown in DMEM containing 10% fetal bovine serum and 2 mM glutamine as described previously [16], [17]. Lipofectamine 2000 (Invitrogen) was used to transfect cells with Tac-chimeras and other plasmids. For the transferrin uptake assay, HeLa cells were starved for 1 h in DMEM with 0.1 BSA (w/v) and Hepes-NaOH (pH 7.5). After washing in cold phosphate buffer saline (PBS) cells were incubated with 1 µg/ml anti-Tac and 0.2 µg/ml transferrin-Alexa488 on ice for 1 h and then chased at 37°C for indicated times by shifting the metal block to a 37°C water bath. Cells were fixed and permeabilized to assess internalization. Dkk1-Flag condition media was generated from Hek293T cells transfected with Dkk1-flag in pCS2. Cells were transfected for 24 h using Lipofectamine2000. Culture media was replaced with fresh serum free DMEM for another 24 h. Media was collected and centrifuged to pellet the cells. Supernatant was stored frozen at −80°C for further use. All the confocal imaging was done using Olympus FluoView FV1000 (Olympus, Center Valley, PA). All the images were acquired using sequential line scanning. All the Z stacked images were analyzed with Fiji (ImageJ) [18]. Photoshop CS5 (Adobe, San Jose CA) was used to modify the TIF images or Adobe Illustrator CS 5 to make a composite figure.

### Surface Labeling and Internalization of Tac

After transfection, cells were labeled with monoclonal anti-Tac antibody, chased at 37°C for 15minutes, and fixed. After blocking in 10% normal goat serum, cells were incubated with mouse anti-IgG-FITC (Jackson Labs). Cells were washed and refixed in 4% paraformaldehyde (PFA) and permeabilized in 0.3% saponin and 10% goat serum, and the cells were stained with mouse anti-IgG-Cy3 (Jackson Labs). Cells on coverslips were mounted on slides for visualization using confocal microscope. Cells co-transfected with LRP6-YFP and Kremen1 WT or mutant tagged with Flag were permeabilized in 0.3% saponin in PBS on ice for 1 min, washed with PBS, and fixed in 4% PFA. After permeabilization in 0.3% saponin, cells were stained with mouse anti-Flag M2 antibody and the secondary antibody was mouse anti-IgG-cy5 (Jackson Labs).

### Sucrose Density Gradient

HeLa cells transfected with a vector for expression of the Tac-Kremen1 cytosolic tail were lysed in TNE buffer (25 mM Tris-HCl (pH 7.5), 150 mM NaCl, 5 mM EDTA, 0.4% Triton X-100, 25 µg/ml Leupeptin, 1 mM phenylmethylsulfonyl fluoride (PMSF), 1 mM benzamidine. Cells were lysed with 40 strokes in dounce homogenizer on ice and passed through a 25 gauge needle (Yamamoto et al. 2008). The homogenate was centrifuged at 12000×g for 8 min at 4°C to obtain post-nuclear supernatant (PNS). The PNS was loaded on the top of 5–40% linear gradient in TNE buffer and centrifuged in a Sorvall TH-641 rotor at 273,000×g for 20 h at 4°C. Fractions of 1 ml were collected from bottom of the gradient and an appropriate amount was resolved on SDS-PAGE for immunoblot using mouse anti-AP-2 α-subunit 100/1, mouse anti-clathrin-HC TD.1, rabbit anti-transferrin receptor CD-71, or mouse anti-Dab2 p96.

### siRNA Treatment

Cells were transfected using oligofectamine with an siRNA targeting the AP-2 α-subunit as described previously [19]. Cells treated with the siRNA were then transfected with Tac-Kremen1 plasmid as described earlier. Cells were then stained with monoclonal anti-Tac or evaluated in the transferrin uptake assay.

### Pitstop2 Inhibition Assay

HeLa cells on coverslips were transfected with Tac-Kremen1 8 to 10 h before the assay. Transfected cells were starved for 45 min in DMEM containing 0.1% FBS at 37°C. Cells were either incubated with 0.1% DMSO or 30 µM Pitstop2 for 15 minutes at 37°C. Transferrin-Alexa488 (50 µg/ml) and the monoclonal anti-Tac antibody were added, and cells were incubated for an additional 15 min at 37°C. Cells were fixed and permeabilized for further analysis.

### Biotinylation

HeLa cells transfected with Tac, Tac-Kremen1, or Tac-Kremen1 (LV-AA) were washed with PBS supplemented with calcium and magnesium salts (PBS^CM+^). Cells were incubated in freshly prepared 1 mg/ml biotin-SS-NHS for 1 h with a change of biotin at 30 min. Cells were quenched in lysine monohydrate and washed with PBS and chased at 37°C for various amounts of time. Cells were then washed on ice with cold PBS^CM+^. Surface labeled biotin was cleaved off by using reduced glutathione, and cells were again washed twice in PBS^CM+^. Cells were lysed in lysis buffer containing 1% Triton X-100 in 25 mM Hepes (pH 7.5), 125 mM potassium acetate, 5 mM magnesium acetate, 2 mM EGTA, 2 mM EDTA, and 50 µM serine protease inhibitor phenylmethanesulfonylfluoride (PMSF) along with complete protease inhibitor cocktail (Roche Applied Science) on ice for 30 min. Debris was removed from lysed cells by centrifugation at 15000 *g* for 10 min at 4°C. Lysates were mixed with 25 µl of packed neutravidin agarose beads and incubated on a rocker for 2 h at 4°C. After washing twice in lysis buffer and PBS, the pellets were resuspended in Laemmli sample buffer. After boiling, the samples were resolved on SDS-polyacrylamide gels and evaluated by immunoblot.

## Results

### Kremen1 is Internalized by Clathrin-mediated Endocytosis

**Figure 1:**
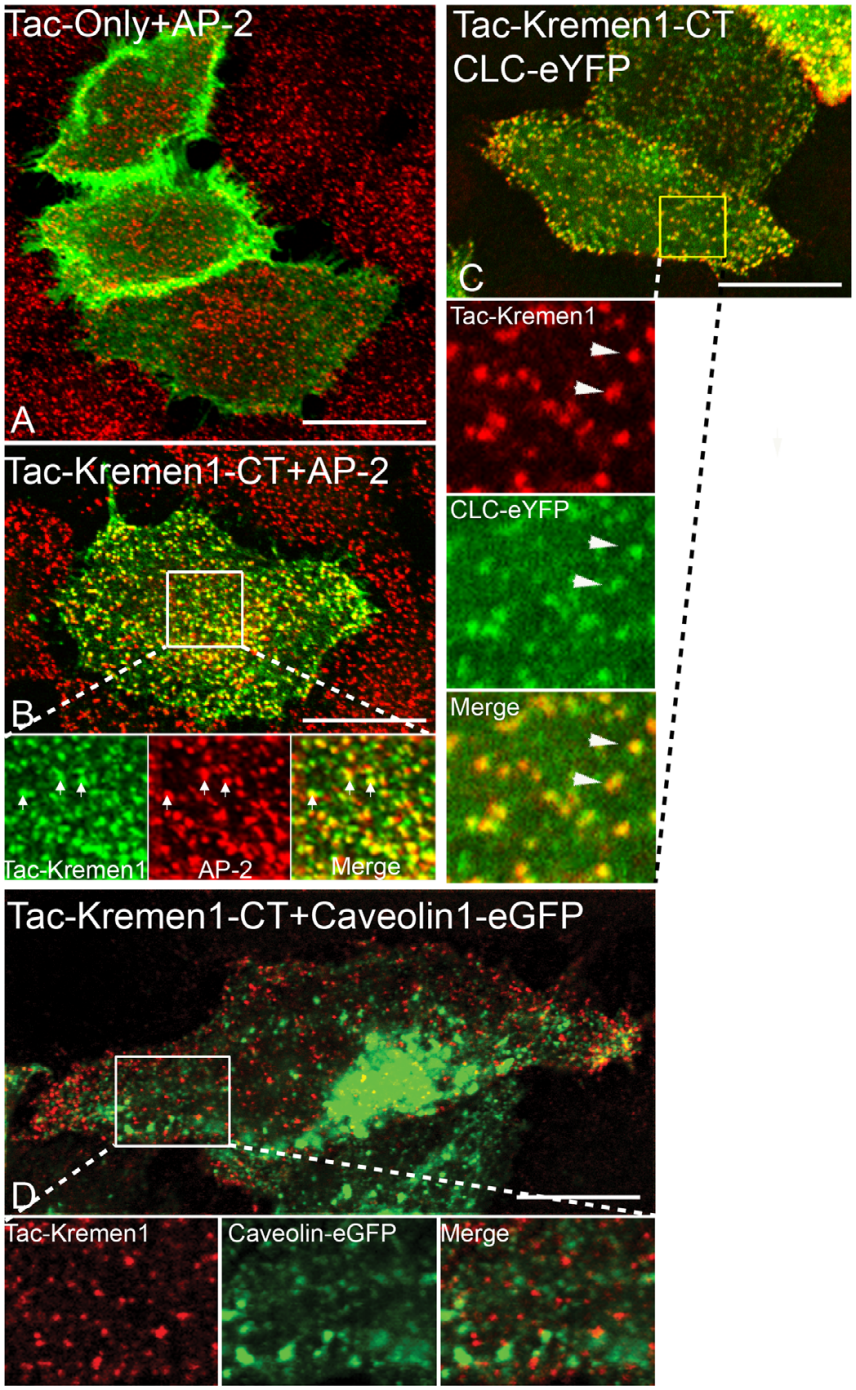
Tac-Kremen1 co-localizes with endocytic proteins. HeLa cells were transfected with (A) Tac alone or (B) Tac-Kremen1. Cells were fixed and permeabilized and stained with mouse monoclonal anti-Tac and Rabbit anti-AP-2 β2 -subunit GD/1. Inset in (B) is zoomed to confirm the co-localization of AP-2 and Tac-Kremen1. HeLa cells were co-transfected with (C) clathrin-LC-eYFP and Tac-Kremen1 or (D) caveolin1-eGFP and Tac-Kremen1. Cells were fixed and permeabilized and incubated with mouse monoclonal anti-Tac antibody. Scale bar 10 µm.

Kremen1 is a kringle domain- containing type I transmembrane protein with no known invertebrate ortholog. This protein is about 35.3% homologous to Kremen2, and was identified in a cDNA screen for Dkk1 binding partners [4], [20]. Kremen proteins co-localize with clathrin in Hek cells [5]. To understand how Kremen1 functions, we made a Kremen1 chimera by fusing the cytosolic tail of Kremen1 (region 414–473) downstream to the extracellular and transmembrane domain of the Tac chain of interleukin-2 receptor α [21] in a mammalian expression vector. In transiently transfected HeLa cells, the Tac domain expressed alone remained on the surface (Fig. 1A). In contrast, the Tac-Kremen1 cytosolic tail fusion (Tac-Kremen1) co-localized with clathrin adaptor protein AP-2 in a distinct punctate pattern (Fig. 1B). HeLa cells co-transfected with Tac-Kremen1 and either caveolin1-eGFP [22] or clathrin LC-eYFP [23] expression plasmids confirmed that Tac-Kremen1 co-localized with a clathrin structure on the cell surface but not with caveloae (Fig. 1C and D). This study suggests that Tac-Kremen1 is trafficked in a clathrin-dependent fashion.

**Figure 2:**
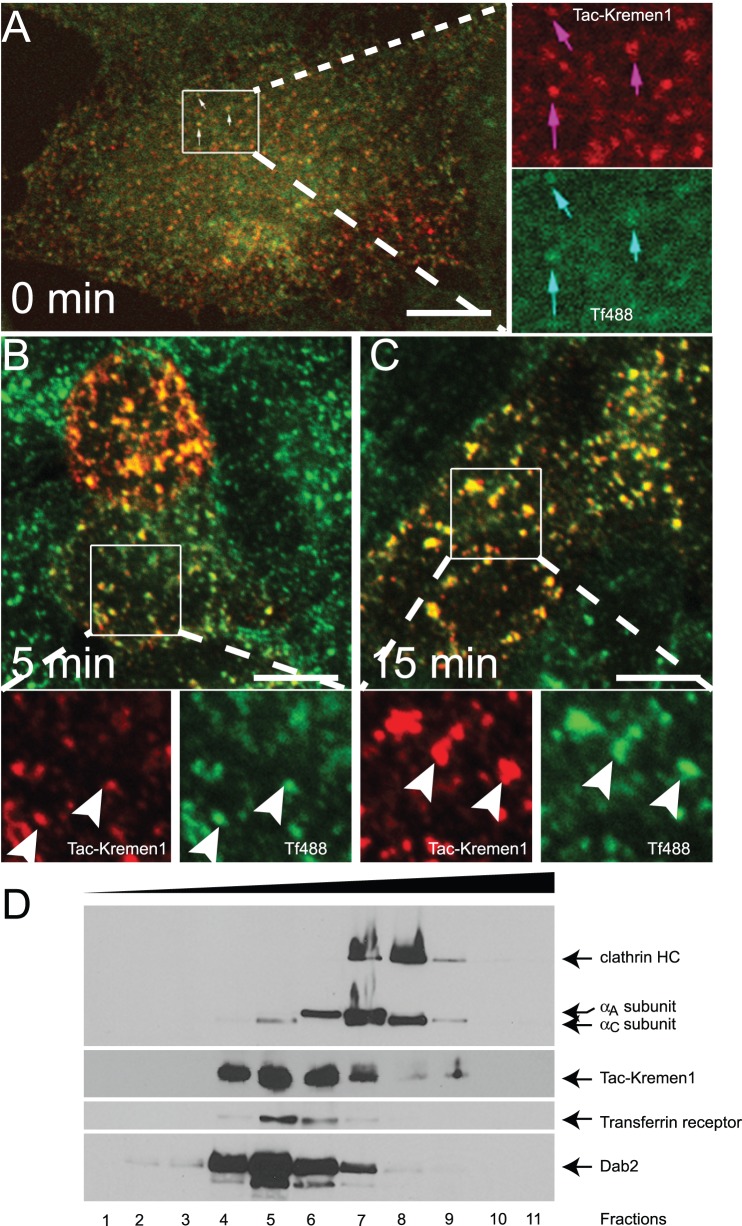
Tac-Kremen1 is trafficked from the cell surface. HeLa cells transfected with Tac-Kremen1 were starved for 1 h in DMEM, 0.1% BSA, and 10 mM Hepes (pH 7.5) and incubated with mouse monoclonal anti-Tac antibody along with transferrin-Alexa488 on ice for 1 h. Cells were then shifted from 4°C to 37°C for (A) 0 min, (B) 5 min, and (C) 15 min, and then were fixed and permeabilized for fluorescence microscopy. Scale bar 10 µm. (D) HeLa cells lystaes expressing Tac-Kremen1 (414–473) were fractionated by sucrose density gradient ultracentifugation and equal portion of the aliquots were probed with anti-Tac antibody. Endogenous clathrin, AP-2 αsubunit, transferrin receptor and Dab2 were identified using mouse anti-clathrin HC TD.1, mouse anti-AP-2 α subunit 100/1, rabbit anti-transferrin receptor CD71 or mouse anti-Dab2 p96.

HeLa cells expressing Tac-Kremen1 were starved for 1 h and incubated along with monoclonal anti-Tac antibody and transferrin-Alexa488 on ice and chased for upto 15 min at 37°C. Tac-Kremen1 and transferrin occupied similar compartments during this phase (Fig. 2A, B, and C). As transferrin is known to be internalized by clathrin-mediated endocytosis, these data further support that Kremen1 is internalized in a clathrin-dependent fashion.

To confirm the association of Tac-Kremen1 with AP-2, HeLa cells were transfected with Tac-Kremen1 and lysates were subjected to linear sucrose density gradient centrifugation. Fractions were resolved by SDS-PAGE and visualized by western blot to confirm the distribution of the proteins (Fig. 2D). Gradients were fractionated from the bottom such that upper fraction numbers correspond to the lower density and lower fraction numbers correspond to higher density of sucrose. Tac-Kremen1 was most abundant in the fractions numbers 4–7. Tac-Kremen1 migrated at a higher molecular mass than either clathrin triskelion (∼650 kDa) or the AP-2 heterotetrameric complex (∼300 kDa) were determined by immunoblots of clathrin heavy chain or α-subunit of AP-2 heterotetrameric complex. The transferrin receptor cargo protein is internalized by clathrin-mediated endocytosis by binding with AP-2 µ2 subunit [24], [25]; the transferrin receptor was present in the fraction numbers 4–7. Thus, the cytosolic fraction of Kremen1 co-fractioned with AP-2 complex, transferrin receptor, and Dab2 in fraction 7, further suggesting that the Kremen1 cytosolic tail participates in clathrin-mediated endocytosis possibly by engaging the AP-2 complex.

Internalization of transmembrane proteins to cellular compartments usually requires sorting motifs present in the cytosolic domain of the transmembrane protein. These sorting motifs are recognized by a member of the family of clathrin-associated sorting adaptors (CLASPs). These adaptors are the major components of clathrin-coated pits on the cell surface [2], [26]. These sorting motifs bind at different surfaces on the AP complexes. Dileucine sorting motifs bind the hemicomplex of α−σ2 subunits [27]–[29]. Kremen1 does not have any typical tyrosine- or dileucine-based sorting motifs.

There are number of ways that CME can be impeded. Various groups have used AP-2 α subunit specific small interfering RNAs (siRNAs) [19], [30], [31], the Dynamin mutant dynK44A [32], and cell-permeable inhibitory reagents such as Dynasore [33] to block the CME from the cell surface. Dynamin is localized in clathrin-coated pits in HeLa cells as shown by fluorescence and electron microscope [34] and plays an important role in the pinching off of the clathrin-coated bud into a clathrin-coated vesicle. Dynasore, a small molecule identified in a chemical screen noncompetitively blocks dynamin GTPase activity, thus blocking dynamin-dependent clathrin-mediated endocytosis [33]. A small molecule chemical inhibitor called pitstop2 also completely inhibits the binding of endocytic proteins such as amphiphysin, AP-180, synaptojanin, and the PH domain of OCRL to clathrin *in vitro* [35]. Pitstop2 also inhibits the trafficking of transferrin receptor from cell surface [35].

**Figure 3:**
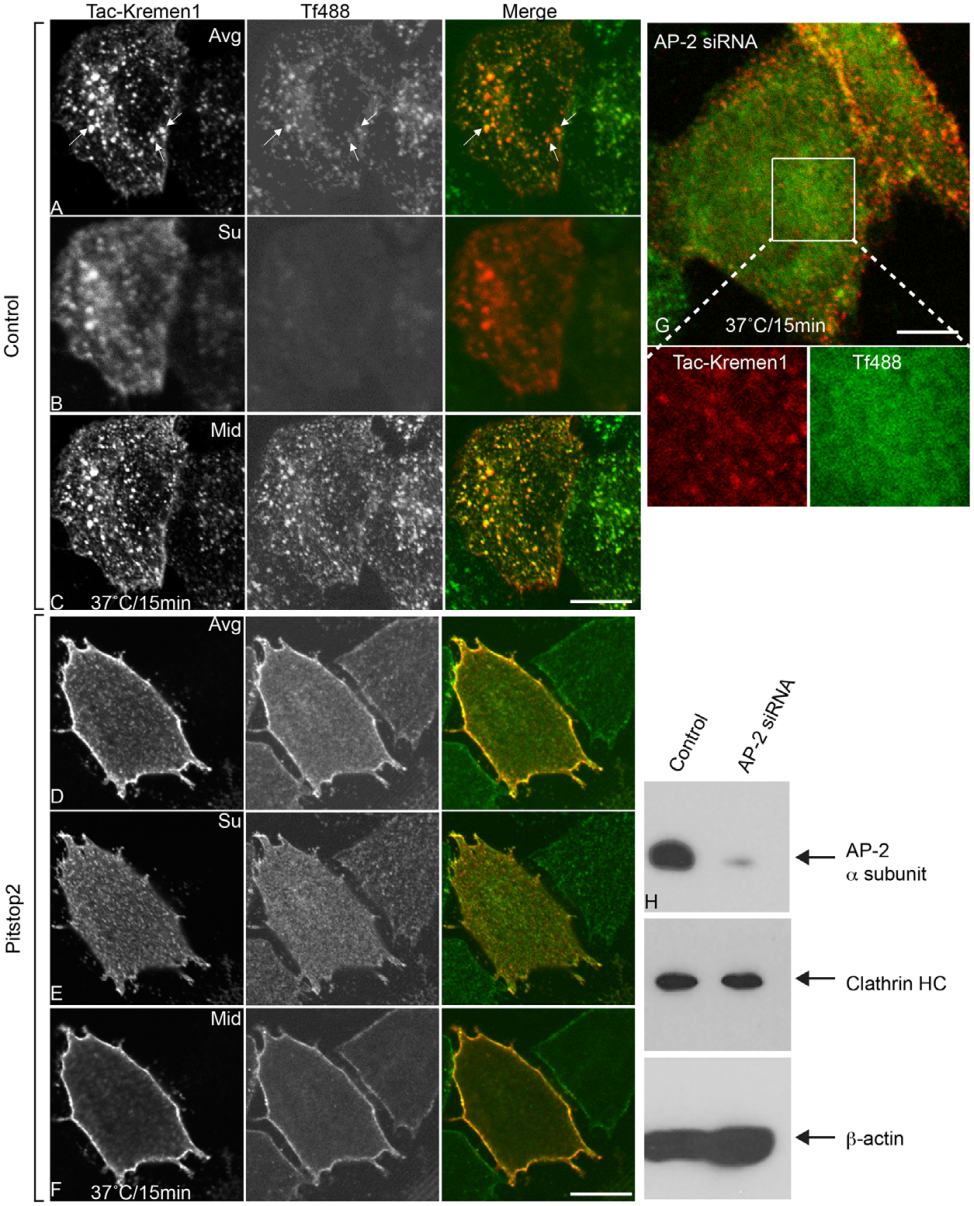
Inhibition of endocytosis of Tac-Kremen1 with CME inhibitors. HeLa cells were transfected with Tac-Kremen1 and then were starved for 45 min in DMEM, 0.1% FBS at 37°C. Starved cells were mixed with 0.1% DMSO (final) (A-C) or 30 µM pitstop2 (final) (D-F) for 15 min at 37°C. Mouse monoclonal anti-Tac and transferrin-Alexa488 (Tf488) were added, and cells were incubated for 15 min at 37°C. Cells were fixed, permeabilized, and stained for confocal microscopy. The Z stack data were stitched by FIJI. (A and D) Representatives of averaged images. (B and E) Single optical sections of surface localization. (C and F) Single optical section of mid sections. (G) HeLa cells were depleted of AP-2 α-subunit by treatment with siRNA and then were transfected with Tac-Kremen1. Cells were starved in DMEM, 0.1% BSA, and 10 mM Hepes (pH 7.5) before incubation on ice with mouse monoclonal anti-Tac and transferrin-Alexa488 for 1 h. The endocytosis assay was performed by shifting the temperature to 37°C for 15 min. Cells were immediately fixed, permeabilized, incubated with anti-mouse-IgG-Cy3 and visualized by confocal microscopy. Scale bar 10 µm. (H) Immunoblot confirming the knockdown of AP-2 α-subunit; levels of clathrin-HC remained unchanged. β-actin was used as a loading control.

In HeLa cells, siRNA-mediated knockdown of AP-2 blocked the endocytosis of the transferrin receptor as well as Tac-Kremen1 at 37°C (Fig. 3G). This suggested that Tac-Kremen1 trafficking depends on AP-2 for its internalization. Depletion of the AP-2 α subunit from these cells was confirmed by immunoblot (Fig. 3H); AP-2 α subunit level were also significantly reduced, however, the level of clathrin heavy chain was unchanged. Similar results were observed when Dynasore was used as a potent inhibitor: Endocytosis of Tac-Kremen1 was inhibited (data not shown). As a number of other pathways depend on dynamin, such as caveolae-dependent and lipid raft-dependent trafficking, inhibition of dynamin did not specifically confrim the route of Kremen1 trafficking. We therefore performed similar experiments after treating cells with pitstop2. HeLa cells incubated with 30 µM pitstop2 showed a complete inhibition of Tac-Kremen1 internalization and transferrin internalization (Fig. 3 D–F), whereas normal trafficking was observed in DMSO treated cells (Fig. 3 A–C). These data indicate that Tac-Kremen1 is internalized via clathrin-mediated endocytosis.

### Identification of the Kremen Sorting Motif

**Figure 4:**
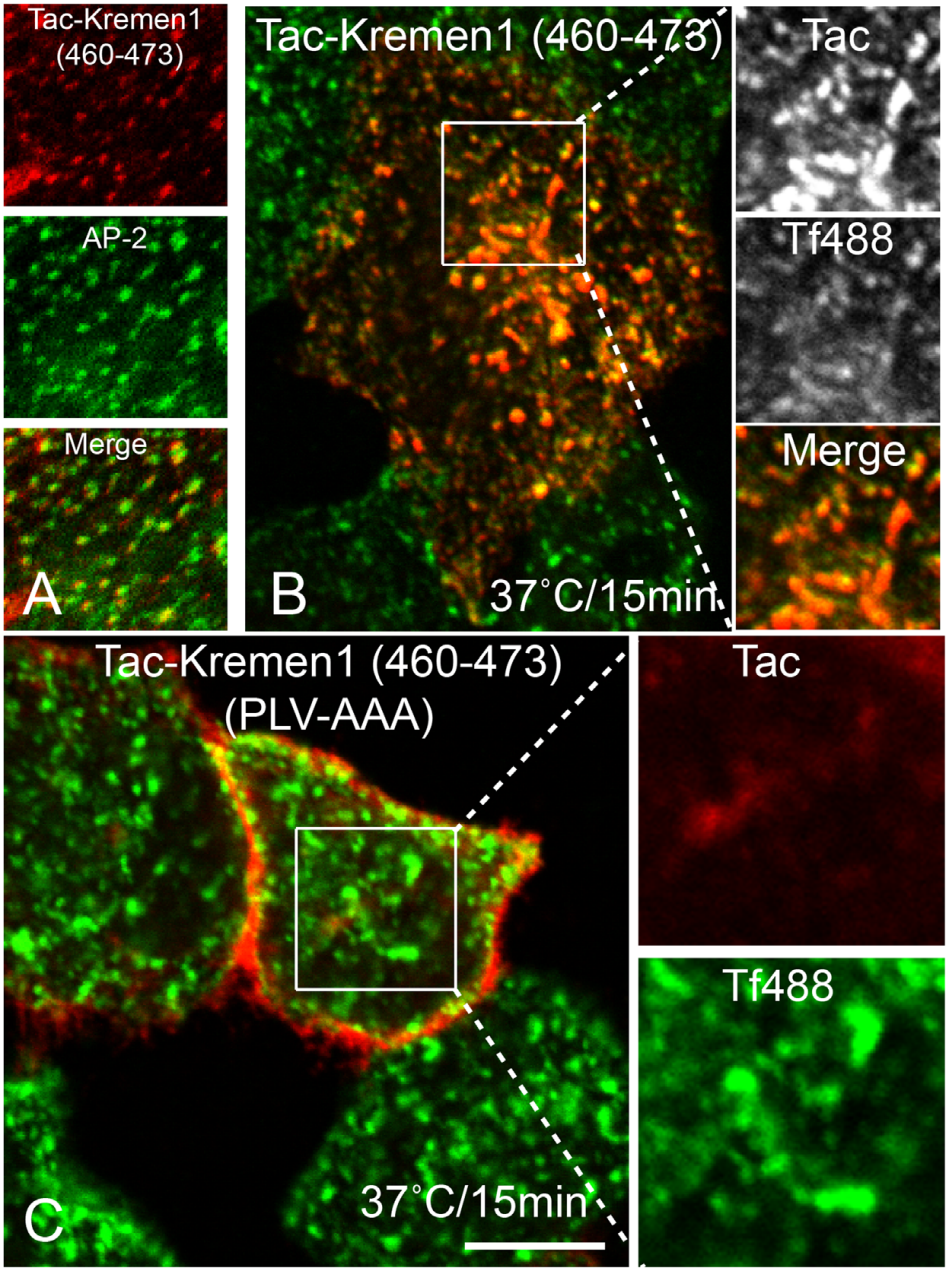
Delineation of endocytic sorting motif in Kremen1. HeLa cells transfected with (A) Tac-Kremen1 (414–460), (C) Tac-Kremen1 (460–473), or (D) Tac-Kremen1 (460–473) (PLV-AAA) were starved for 1 h in DMEM, 0.1% BSA, and 10 mM Hepes (pH 7.5). (A, C, and D) Cells were incubated with mouse monoclonal anti-Tac and transferrin-Alexa488 on ice for 1 h and chased at 37°C for 15 min. (B) HeLa cells transfected with Tac-Kremen1 (460–473) were fixed and permeabilized and incubated with mouse monoclonal anti-Tac or rabbit anti-AP-2 β-subunit GD/1 to confirm the co-localization by confocal microscopy. Scale bar 10 µm.

**Figure 5:**
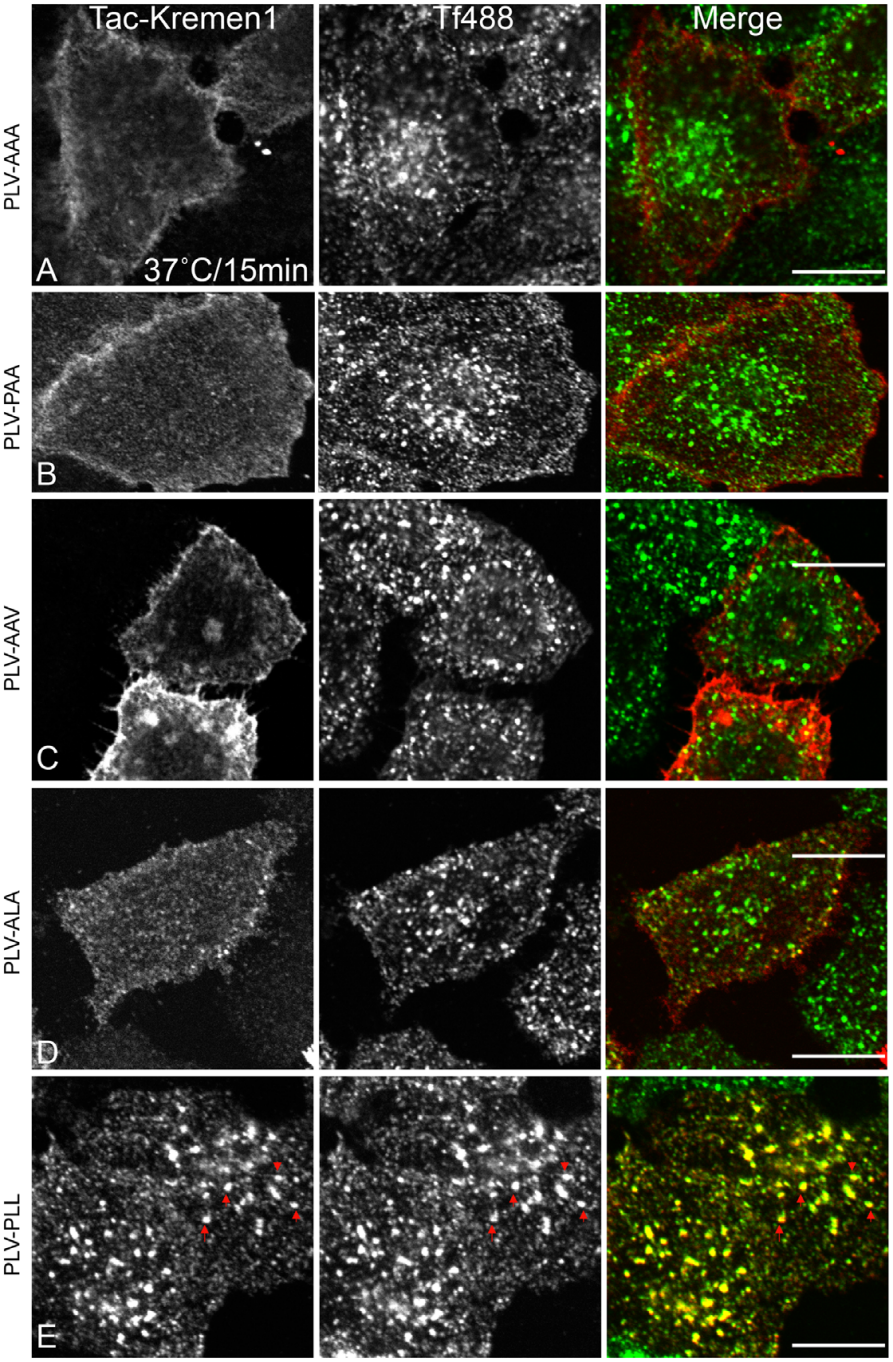
Delineation of endocytic sorting motif in Kremen1. HeLa cells were transfected with (A) Tac-Kremen1 (PLV-AAA), (B) Tac-Kremen1 (PLV-PAA), (C) Tac-Kremen1 (PLV-AAV), (D) Tac-Kremen1(PLV-ALA), or (E) Tac-Kremen1 (PLV-PLL). Transfected cells were starved in DMEM, 0.1% BSA, and 10 mM Hepes (pH 7.5) at 37°C. After washing with cold PBS, cells were incubated with mouse monoclonal anti-Tac and transferrin-Alexa488. Cells were chased at 37°C for 15 minutes. Cells were fixed, permeabilized, and stained with anti-mouse-IgG-Cy3. Scale bar 10 µm.

Internalization of transmembrane proteins to specific cellular compartments usually requires sorting motifs present in the cytosolic domain of the transmembrane protein. These sorting motifs are recognized by a member of the family of clathrin-associated sorting adaptors (CLASPs). The cytosolic tail of Kremen1 does not have typical tyrosine- or dileucine-based sorting motifs, although we did identify a WXXF sequence that might bind to the α-appendage of AP-2 as previously reported for many CLASPs [36], [37]. Mutating either tryptophan to alanine (data not shown) or phenylalanine to alanine of WWXF (Fig. S1A) did not interfere with the trafficking of Kremen1, indicating that other trafficking motifs must be present in the cytosolic tail of Kremen1. We created two Kremen1 truncated mutants containing stop codons after 440 and 460 of Tac-Kremen1. Neither of these mutants internalized normally (Fig. S1B and C). Thus, the sorting sequence was isolated to be present in the region after position 460. We created a Tac-chimera containing the residues 460 to 473 of Kremen1 (Tac-Kremen1-C2). This transplantable chimera co-localized with AP-2-containing punctate spots (Fig. 4A) and was internalized normally along with transferrin (Fig. 4B). This data confirmed that the region from 460 to 473 was necessary for Kremen1 trafficking. We used alanine scanning mutagenesis to mutate three regions, QQD (463–465), DRN (466–468), and PLV (469–471), to alanine. Changes of QQD or DRN to AAA in the context of Tac Kremen1-C2 did not affected trafficking (Fig. S2A and S2B), whereas the PLV to AAA mutation resulted in impaired Tac-Kremen1 trafficking (Fig. 4C). When similar mutagenesis was performed in the cytosolic tail, but in the context of a larger region of the Kremen1 cytosolic tail, endocytosis was completely inhibited (Fig. 5A). Mutation of PLV to PAA or AAV completely blocked internalization (Fig. 5B and C); mutation of PLV to ALA, impaired but did not completely inhibit endocytosis of the Tac-Kremen1 chimera (Fig. 5D). The mutation of this construct back to PLL rescued its internalization (Fig. 5E). These data indicate that two amino acids, LV, at positions 470 and 471 of the full-length Kremen1, are essential for its endocytosis.

**Figure 6:**
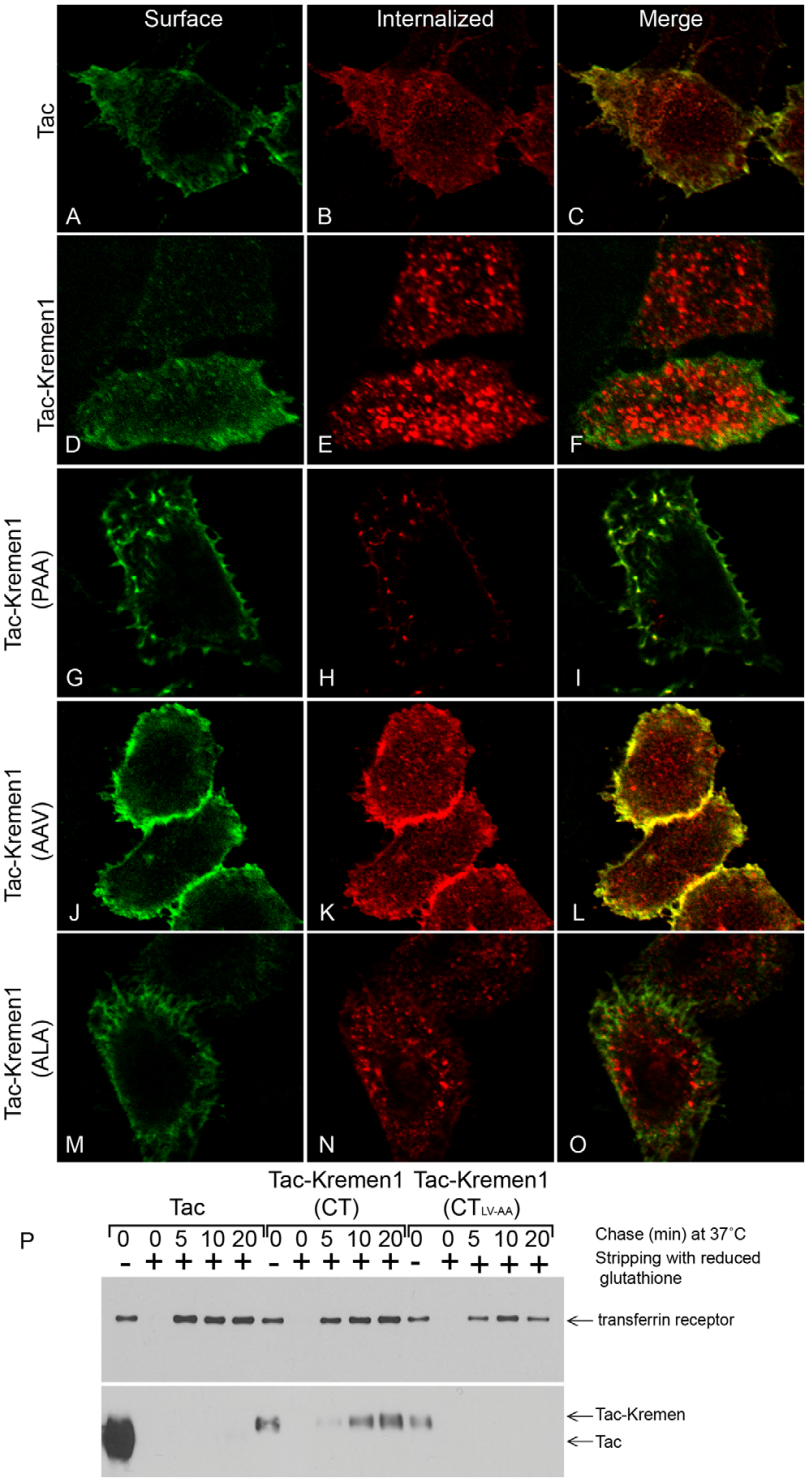
Delineation of endocytic sorting motif in Kremen1. HeLa cells were transfected with (A–C) Tac alone, (D–F) Tac-Kremen1, (G–I) Tac-Kremen1 (PLV-PAA), (J–L) Tac-Kremen1 (PLV-AAV), or (M–O) Tac-Kremen1 (PLV-ALA) and were incubated with mouse monoclonal anti-Tac antibody and chased for 15 minutes at 37°C. (A, D, G, J, and M) Cells were fixed and blocked without permeabilization and labeled with anti-mouse IgG-FITC to visualize surface Tac population. (B, E, H, K, and N) Cells were refixed in 4% PFA, permeabilized, and stained with anti-mouse IgG-Cy3 to visualize the internalized population of Tac. (C, F, I, L, and O) Merge images corresponding to internalized population. Scale bar 10 µm. (P) HeLa cells were transfected with Tac alone, Tac-Kremen1, or Tac-Kremen1 (PLV-PAA), surface labeled with biotin, and chased for 0–20 min at 37°C. Cells were washed to strip surface labeled biotin to confirm the internalized population. Cells were detergent solubilized and after centrifugation incubated with NeutrAvidin-agarose for 2 h at 4°C. Beads were collected after centrifugation, washed in lysis buffer, and resuspended in SDS-sample buffer. After SDS-PAGE protein separation, proteins were transferred onto nitrocellulose membrane. Portions of the blot were stained with (top panel) rabbit monoclonal anti-CD71 transferrin receptor and (bottom panel) mouse monoclonal anti-CD25 IL2 receptor antibody.

To further validate the mechanism of Kremen1 internalization, HeLa cells were transiently transfected with Tac, Tac-Kremen1, Tac-Kremen1 (PLV-PAA), Tac-Kremen1 (PLV-AAV), or Tac-Kremen1 (PLV-ALA). Transfected cells were incubated with anti-Tac antibody on ice and chased for 15 min at 37°C. Fixed and non-permeabilized cells were labeled with mouse IgG-Alexa488 to detect Tac. The washed cells were refixed in paraformaldehyde and permeabilized and incubated with mouse anti-IgG-Cy3 to quantitate the internalized Tac population. These data confirmed that the Tac and the PAA and AAV Tac-Kremen1 mutants remained on the surface (Fig. 6 A, G and J) but that Tac-Kremen1 was internalized normally and the Tac-Kremen1 (ALA) was slowly internalized (Fig. 6 E and N).

To confirm the trafficking of Kremen1 biochemically, HeLa cells transfected with Tac, Tac-Kremen1, or Tac-Kremen1 (PAA) were surface labeled with biotin. After internalization at 37°C for different time periods, cell surface biotin was cleaved off by non-permeable reduced glutathione. Cells were lysed and incubated with neutrAvidin-agarose beads. After SDS-PAGE, resolved proteins were transferred on the nitrocellulose membrane and probed with either anti-CD71 transferrin receptor antibody or anti-Tac antibody (Fig. 6P). Only wild type Tac-Kremen1 was internalized normally; Tac and the Tac-Kremen1 (PAA) mutant remained on the cell surface (Fig. 6 D–I). The control transferrin receptor was internalized normally in all the treatment conditions. This data fully supports the idea that the LV sequence is the sorting signal motif of Kremen1.

### Conserved Sequence in Kremen1

**Figure 7:**
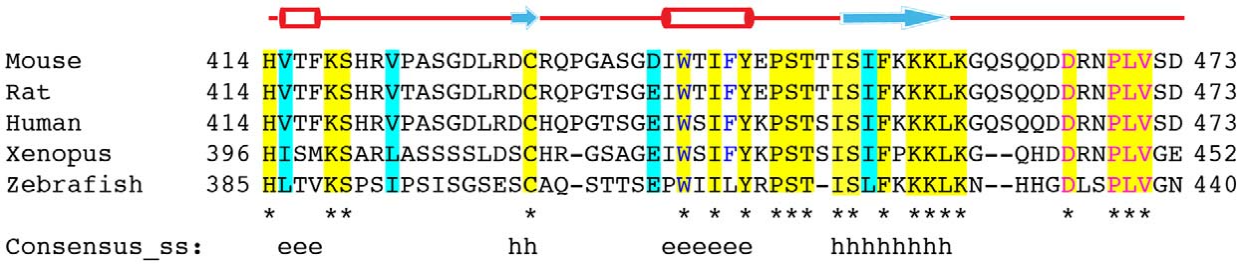
Alignment of cytosolic tail of Kremen1. Amino acid alignment of Kremen1 cytosolic tails of mouse (NM_032396), rat (NM_053649), human (NM_00103957), *Xenopus laevis* (NM_001088676), and zebrafish (NM_001114917). Conserved residues are shaded in yellow and marked with asterisks and identical residues are indicated in blue.

Upon amino acid alignment of cytosolic tails of human (Q96MU8), mouse (Q99N43), rat (Q924S4), *Xenopus* (Q90Y90), and zebrafish (F1QW1Z) Kremen1 proteins, we identified a well conserved C-terminus LV sequence (Fig. 7); these amino acids are also present in the cytosolic tail of Kremen2 protein. This suggested that in some type I transmembrane receptors an atypical dileucine signal, LV, LL, or LI, is important for its internalization by clathrin-mediated endocytosis. In the Kremen1 alignments, there is a conserved aspartic acid residue at position 466 preceding the LV so the conserved signal may be DXXXLV. Mutation of Asp^466^ did not interfere with the trafficking of Kremen1 suggesting that this aspartic acid residue found in many other receptors [38], is not an essential feature of this sorting signal.

### Kremen1 LV-AA Mutant Does not Co-localize with Known Markers

**Figure 8:**
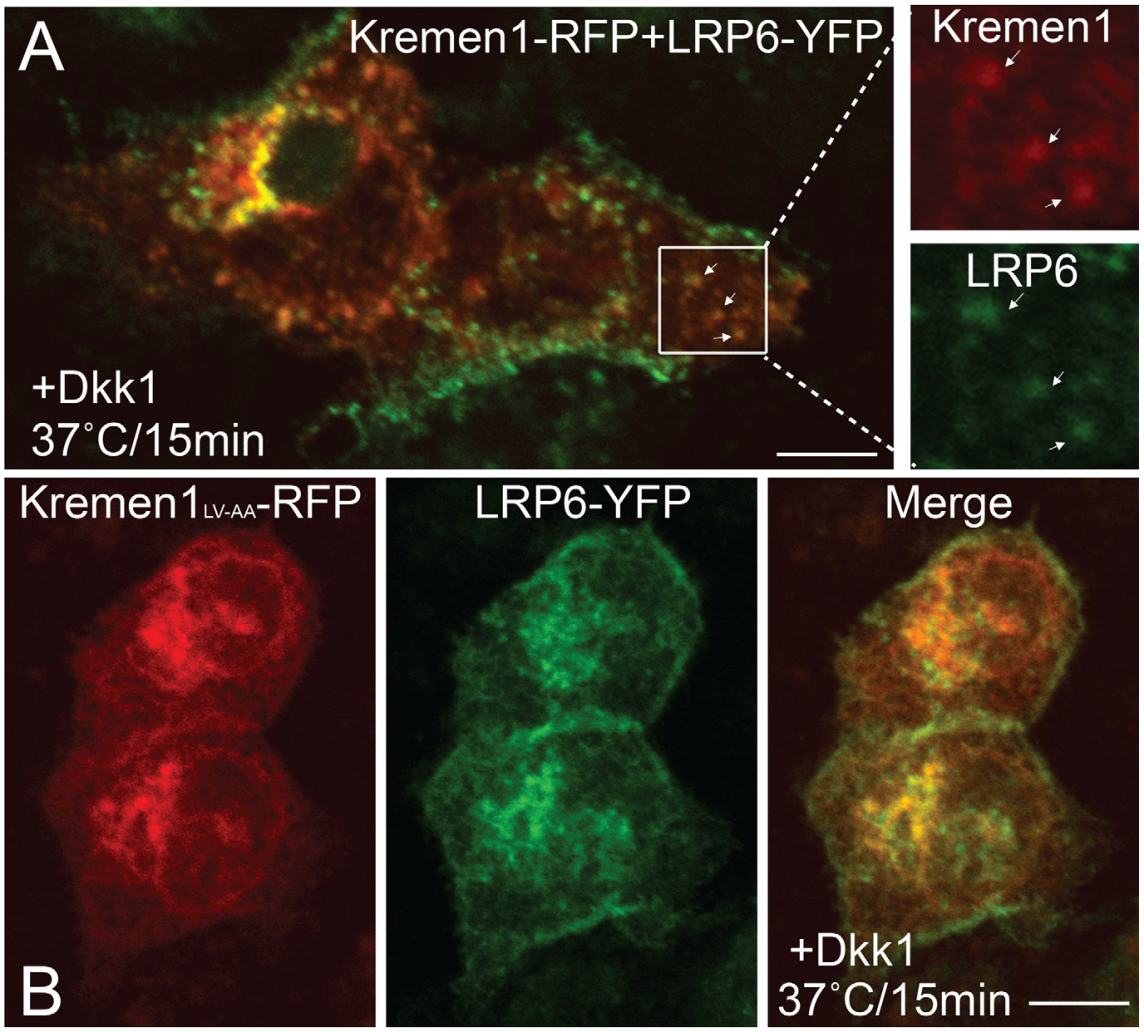
Full-length Kremen1 co-localizes with LRP6. HeLa cells were co-transfected with (A) LRP6-eYFP and Kremen1-mRFP WT or (B) LRP6-eYFP and Kremen1_LV-AA_-mRFP mutant. Cells were incubated in presence of pCS2-Dkk1-flag for 60 min at 37°C. Cells were permeabilized on ice before fixation for 1 min on ice; after washing cells were fixed to visualize under confocal microscopy. Scale bar 10 µm.

The full-length flag-tagged Kremen1 has similar endocytic properties as Tac-Kremen1. It co-localizes with transferrin after a chase at 37°C, whereas flag-tagged mutant (LV-AA) does not co-localize with transferrin suggesting that LV is the main sorting signal for Kremen family members (Fig. S3). When Kremen1-mRFP was co-transfected with LRP6-YFP the two were observed in the same compartment in the presence of Dkk1 in the media (Fig. 8A); the mutant did not co-localize with LRP6 (Fig. 8B). In the absence of Dkk1, the wild-type Kremen1 did not co-localize with LRP6 (Data not shown). These results suggest that down-regulation of LRP6 by Dkk1 requires the internalization of Kremen1 and the LV sorting signal.

## Discussion

Early developing mouse embryos express *krm1* and *krm2* mRNAs encoding Kremen1 and Kremen2, respectively [20]. The transcripts of *krm1* and *krm2* are expressed in early developmental stages in mouse in sensory organs, lung, and heart [39], [40]. Morpholino-mediated down-regulation of Kremen1 and Kremen2 in *Xenopus* led to embryonic developmental defects in the head, which phenocopies the effect of Dkk1 down-regulation [40]. These results suggest that Kremen is a Wnt inhibitor that in turn regulates the anterioposterior patterning of the central nervous system [40].

Kremens 1 and 2 are high-affinity type I transmembrane receptors of Dkk1. Dkk1 is a secreted glycoprotein that negatively regulates Wnt/β-catenin signaling [4], [39] by binding to LRP6, a co-receptor of Wnt ligand, prior to internalization by clathrin-mediated endocytosis [5]. Formation of a ternary complex of Dkk1, LRP6, and Kremen results in inhibition of Wnt signaling. Here we report the mechanism by which Kremen1 is internalized. Our data indicate that Kremen1 internalization requires clathrin and AP-2. siRNA-mediated inhibition of AP-2 expression in HeLa cells perturbed the endocytosis of a Tac-Kremen1 chimera. Previously it was shown that inhibition of AP-2 α subunit or clathrin heavy chain expression impaired the ability of transferrin receptor endocytosis while other receptors not dependent on AP-2 for endocytosis were unaffected [30], [41]. A similar defect in Kremen1 internalization was also observed after treatment of cells with pitstop 2, a small molecule inhibitor of clathrin-mediated endocytosis or using Dynasore [35]. Pitstop 2 has been shown to inhibit the internalization of subtilisin-like proprotein convertase family member proprotein convertase 7 (PC7) from the cell surface by blocking the clathrin terminal domain binding site [42]. These data support the previously reported studies that the internalization of Kremen1 requires clathrin and AP-2 [43].

A number of sorting motifs have been identified that selectively direct cargo to clathrin-coated pits. These sorting motifs are either tyrosine based YXXØ (where XX is any amino acids and Ø is a bulky hydrophobic amino acid) or the dileucine-containing [D/E]XXXL[L/I], and are recognized by adaptor proteins AP-1, AP-2, and AP-3 [2]. Other sorting motifs such as FXNPXY bind to the phospho-tyrosine binding domain (PTB) of adaptor proteins such as Dab2 and ARH [12], [44]. Dileucine sorting motifs are usually present in mammalian proteins that shuttle from cell surface to endosomal-lysosomal compartments or trans-Golgi network to the endosomal compartment [2]. Kremen1 has none of these motifs; rather, our study suggests that an atypical leucine-valine pair in the cytoplasmic tail of Kremen1 is essential for its internalization.

In a recent study Howe’s group suggested that Dab2 recruits LRP6 for clathrin-mediated endocytosis [45]. Dab2 was first identified as a tumor suppressor and a negative regulator of Wnt/β-catenin signaling and is an endocytic CLASP required for sorting of LDLR by recognizing FXNPXY sorting signal through its N-teminal PTB domain [46], [47]. It is present on the clathrin-coated structure and physically interacts with α subunit of AP-2 using short peptide motifs DPF and FXDXF [12], [48]. Dab2 contains several smaller modular motifs and domains, thus allowing Dab2 to interact with various proteins at the clathrin-coated bud on the cell surface [12]. Interestingly, internalization of LRP6 by Dab2 by a clathrin-dependent pathway requires the phosphorylation of LRP6 by casein kinase 2 (CK2) at S1579 thus downregulating Wnt/β-catenin signaling [45]. Our data, and that of Jiang et al. (2012), suggest that LRP6 internalization by clathrin-mediated endocytosis occurs by a different mechanism that depends on spatial needs. Spatiotemporal down-regulation of Wnt/β-catenin is important in lung development and morphogenesis.

Our findings are in agreement with the previously published results by Yamamoto et al. [5] and Mao et al. [4] that clathrin-mediated down-regulation of LRP6 by Dkk1 depends on the presence of Kremen. However, physiological function of Kremen1 on the cell surface may not require an intracellular domain, but the sorting signal present in the intracellular domain might help Kremen1 in its internalization from the cell surface by clathrin-mediated endocytosis. It has been reported that both physical and functional interactions between Dkk1 and Kremen require the extracellular domain of Kremen. Ablation of any part of the extracellular domain of Kremen2 inhibits its interaction with Dkk1 (Mao et al. 2002). However, our data strongly suggest that the atypical leucine-valine pair present in the cytosolic tail is critical for its internalization by clathrin-mediated endocytosis from the cell surface. In agreement with previously published work of Mao et al. (2002) and Yamamoto et al. (2006) and of our data suggests that possibly the biochemical activity of the Kremen1 may well be exerted upon the initiation of ternary complex formation between Dkk1, LRP6 and Kremen on the cell surface, even before the internalization of Kremen1 by clathrin-mediated endocytosis. Mao et al. [4] suggested that in the presence of Dkk1, physical interaction between LRP6 and Kremen does not require the Kremen cytosolic tail. However, their data predicts that Kremen2 mediates LRP6 internalization in the presence of Dkk1 within 5 minutes of chase at 37°C, suggesting that the cytosolic tail of Kremen has the potential to internalize by clathrin-mediated endocytosis. Yamamoto et al. [5] also showed the clathrin-mediated internalization of LRP6 in presence of Dkk1 when Flag-Kremen2 was co-expressed in HeLa cells. We found that the LV sequence present in the cytosolic tail of Kremen1 is required for its clathrin-mediated internalization. Kremen1 has a DXXXLV sequence, but mutation of the acidic residue did not alter internalization. In addition, this residue is not found in Kremen2 suggesting acidic residue is not necessary for its function. Kremen2 also contains the LV sequence found in Kremen1. The cytosolic domain of Kremen2 also has an atypical dileucine sequence near the transmembrane domain. Further experiments will be required to determine whether the LV sequence present in the cytosolic tail of Kremen2 plays the same role in endocytosis as it does in the tail of Kremen1. Kremen1 may adopt an extended conformation to allow access of the adaptor protein to the LV residues.

## Supporting Information

Figure S1 **Delineation of endocytic sorting motif in Kremen1.** HeLa cells transfected with (A) Tac-Kremen1 (F444A), (B) Tac-Kremen1 (414–440), or (C) or Tac-Kremen1 (414–460) were starved for 1 h in DMEM, 0.1% BSA, and 10 mM Hepes (pH 7.5). Cells were incubated with mouse monoclonal anti-Tac and transferrin-Alexa488 on ice for 1 h and chased at 37°C for 15 min. Cells were fixed and permeabilized and incubated with anti-mouse IgG-Cy3 to confirm the co-localization by confocal microscopy. Scale bar 10 µm.
(TIF)

Figure S2 **Delineation of endocytic sorting motif in Kremen1.** HeLa cells transfected with (A) Tac-Kremen1 (QQD-AAA) or (B) Tac-Kremen1 (DRN-AAA) were starved for 1 h in DMEM, 0.1% BSA, and 10 mM Hepes (pH 7.5). Cells were incubated with mouse monoclonal anti-Tac and transferrin-Alexa488 on ice for 1 h and chased at 37°C for 15 min. Cells were fixed and permeabilized and incubated with anti-mouse IgG-Cy3 to confirm the co-localization by confocal microscopy. Scale bar 10 µm.
(TIF)

Figure S3 **Wild type (WT) full-length Kremen1 colocalizes with transferrin.** HeLa cells transfected with flag-tagged Kremen1 (WT) (A–C), or (PLV-PAA) (D–F) were starved for 1 h in DMEM, 0.1% BSA, and 10 mM Hepes (pH 7.5). Cells were incubated with transferrin-Alexa488 on ice for 1 h and chased at 37°C for 15 min. Cells were fixed and permeabilized and incubated with mouse anti-Flag M2 followed by washing and staining with anti-mouse IgG-Cy3 to confirm the co-localization by confocal microscopy.
(TIF)
