# Supplementary figures and images for: High-Affinity Dkk1 Receptor Kremen1 Is Internalized by Clathrin-Mediated Endocytosis
Source: PLoS One. 2012 Dec 14;7(12):e52190. doi: 10.1371/journal.pone.0052190 (PMC3522622; doi:10.1371/journal.pone.0052190)

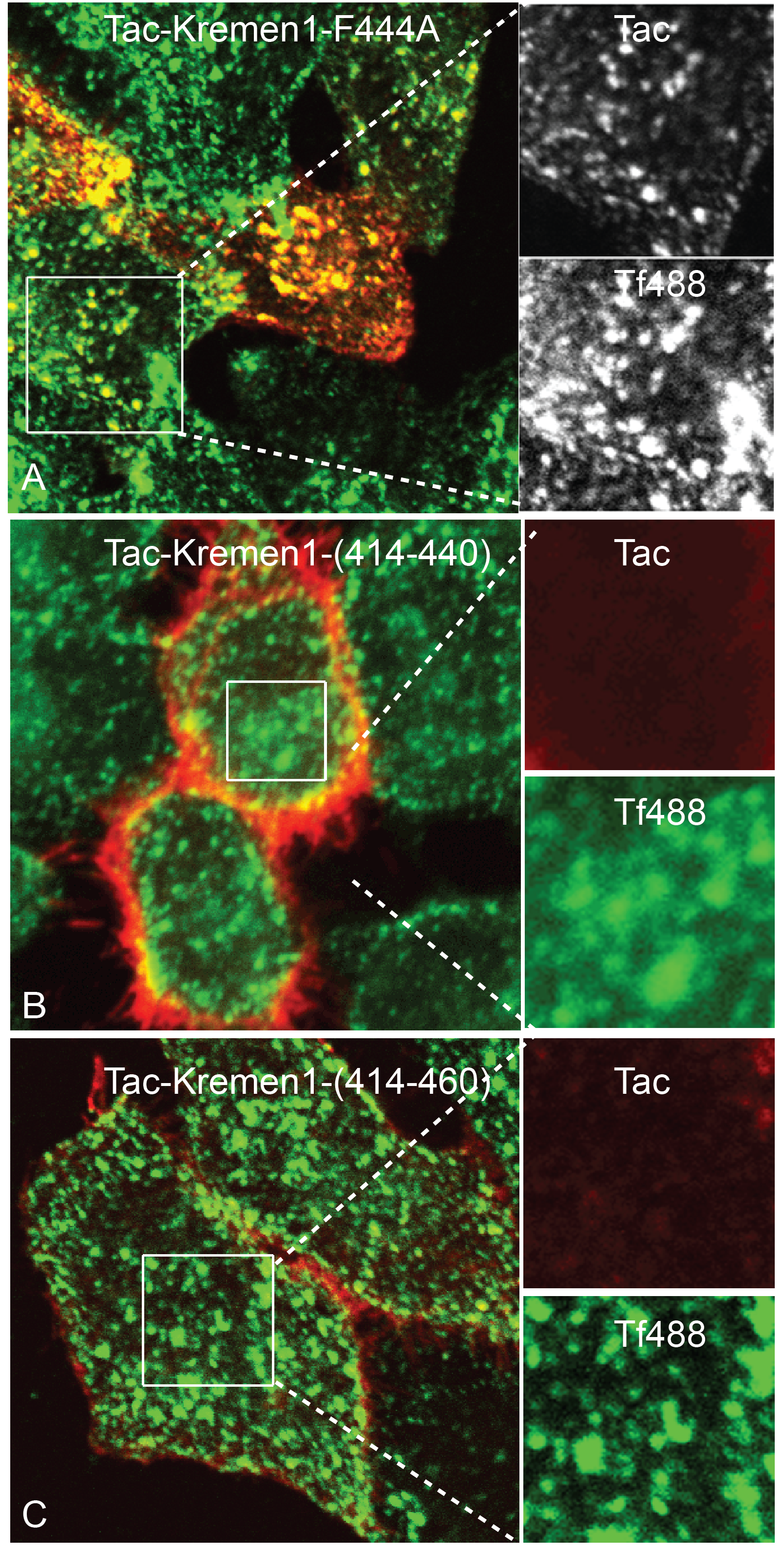

Supplement: Figure S1 — Delineation of endocytic sorting motif in Kremen1. HeLa cells transfected with (A) Tac-Kremen1 (F444A), (B) Tac-Kremen1 (414–440), or (C) or Tac-Kremen1 (414–460) were starved for 1 h in DMEM, 0.1% BSA, and 10 mM Hepes (pH 7.5). Cells were incubated with mouse monoclonal anti-Tac and transferrin-Alexa488 on ice for 1 h and chased at 37°C for 15 min. Cells were fixed and permeabilized and incubated with anti-mouse IgG-Cy3 to confirm the co-localization by confocal microscopy. Scale bar 10 µm. (TIF) [file pone.0052190.s001.tif]

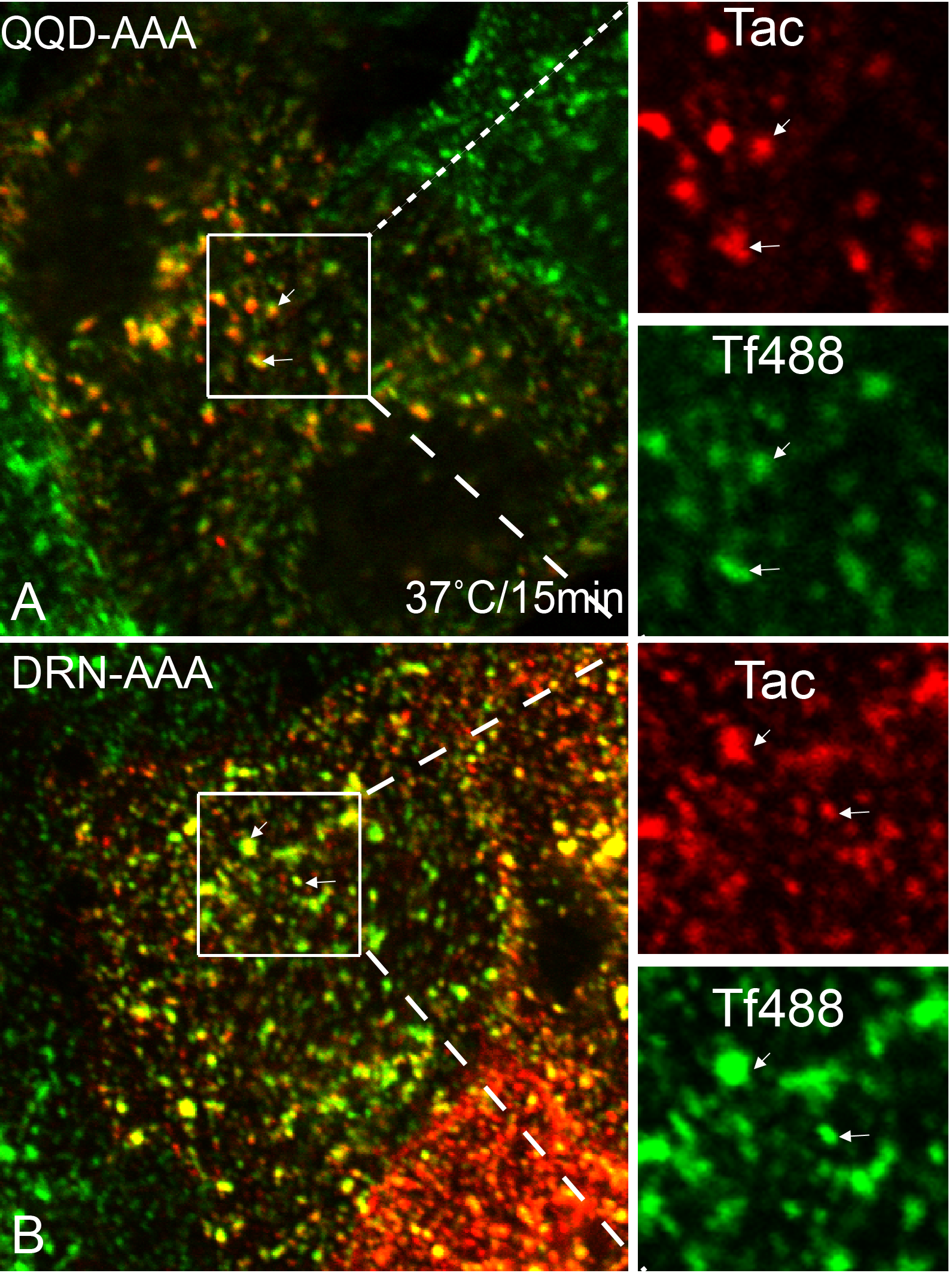

Supplement: Figure S2 — Delineation of endocytic sorting motif in Kremen1. HeLa cells transfected with (A) Tac-Kremen1 (QQD-AAA) or (B) Tac-Kremen1 (DRN-AAA) were starved for 1 h in DMEM, 0.1% BSA, and 10 mM Hepes (pH 7.5). Cells were incubated with mouse monoclonal anti-Tac and transferrin-Alexa488 on ice for 1 h and chased at 37°C for 15 min. Cells were fixed and permeabilized and incubated with anti-mouse IgG-Cy3 to confirm the co-localization by confocal microscopy. Scale bar 10 µm. (TIF) [file pone.0052190.s002.tif]

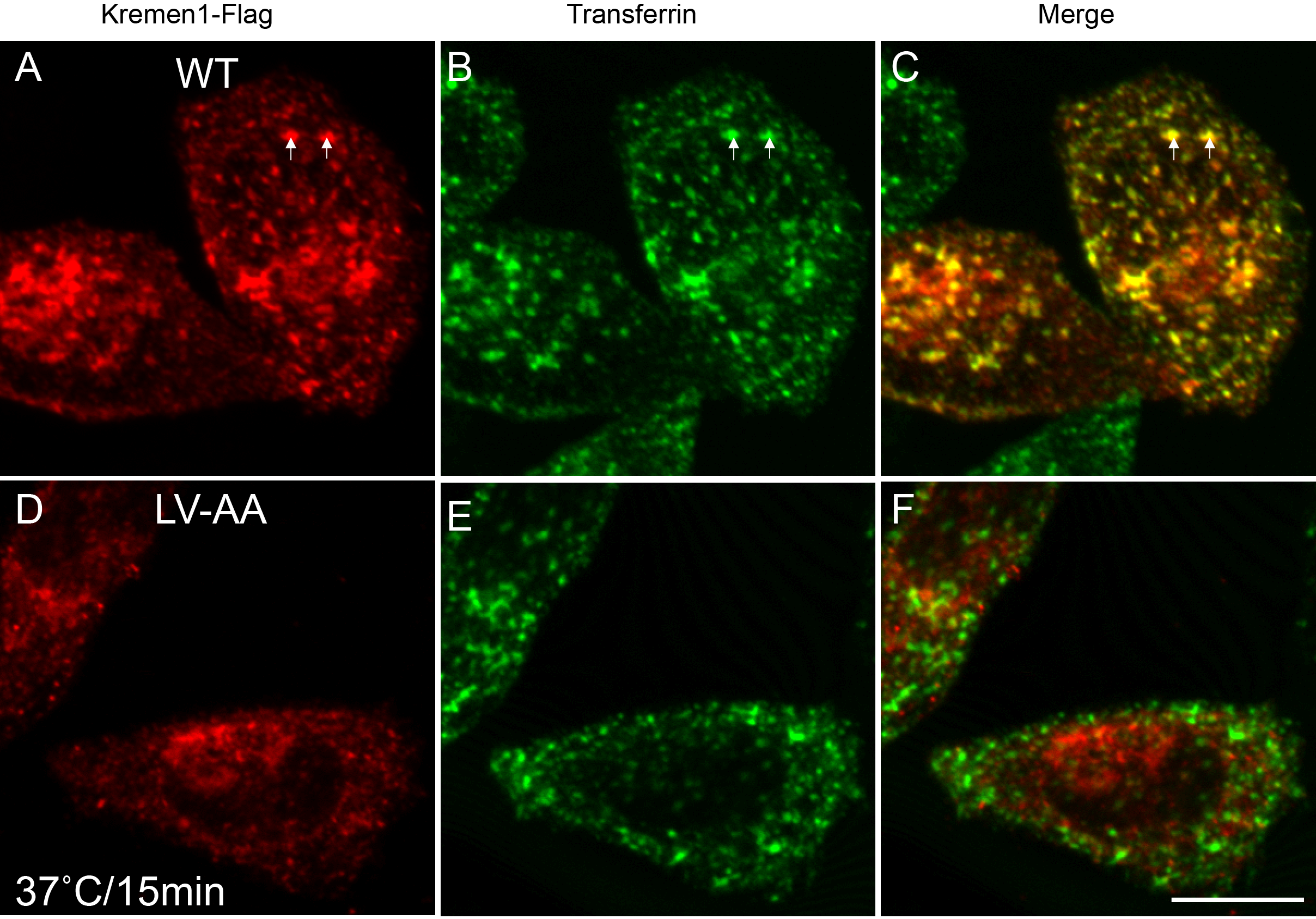

Supplement: Figure S3 — Wild type (WT) full-length Kremen1 colocalizes with transferrin. HeLa cells transfected with flag-tagged Kremen1 (WT) (A–C), or (PLV-PAA) (D–F) were starved for 1 h in DMEM, 0.1% BSA, and 10 mM Hepes (pH 7.5). Cells were incubated with transferrin-Alexa488 on ice for 1 h and chased at 37°C for 15 min. Cells were fixed and permeabilized and incubated with mouse anti-Flag M2 followed by washing and staining with anti-mouse IgG-Cy3 to confirm the co-localization by confocal microscopy. (TIF) [file pone.0052190.s003.tif]
